# The Origin of Discrepancies between Predictions and Annotations in Intrinsically Disordered Proteins

**DOI:** 10.3390/biom13101442

**Published:** 2023-09-25

**Authors:** Mátyás Pajkos, Gábor Erdős, Zsuzsanna Dosztányi

**Affiliations:** Department of Biochemistry, ELTE Eötvös Loránd University, Pázmány Péter Stny 1/c, H-1117 Budapest, Hungary; matyas.pajkos@ttk.elte.hu (M.P.); gabor.erdos@ttk.elte.hu (G.E.)

**Keywords:** intrinsically disordered proteins, intrinsically disordered regions, disorder prediction methods, AlphaFold2, IUPred, DisProt, disorder to order transition, molten globule, flexible linker

## Abstract

Disorder prediction methods that can discriminate between ordered and disordered regions have contributed fundamentally to our understanding of the properties and prevalence of intrinsically disordered proteins (IDPs) in proteomes as well as their functional roles. However, a recent large-scale assessment of the performance of these methods indicated that there is still room for further improvements, necessitating novel approaches to understand the strengths and weaknesses of individual methods. In this study, we compared two methods, IUPred and disorder prediction, based on the pLDDT scores derived from AlphaFold2 (AF2) models. We evaluated these methods using a dataset from the DisProt database, consisting of experimentally characterized disordered regions and subsets associated with diverse experimental methods and functions. IUPred and AF2 provided consistent predictions in 79% of cases for long disordered regions; however, for 15% of these cases, they both suggested order in disagreement with annotations. These discrepancies arose primarily due to weak experimental support, the presence of intermediate states, or context-dependent behavior, such as binding-induced transitions. Furthermore, AF2 tended to predict helical regions with high pLDDT scores within disordered segments, while IUPred had limitations in identifying linker regions. These results provide valuable insights into the inherent limitations and potential biases of disorder prediction methods.

## 1. Introduction

The functional form of proteins encompasses a variety of structural states that include not only globular domains but also intrinsically disordered proteins (IDPs) [1]. IDPs possess highly flexible regions (IDRs) or entire sequences that lack a stable fold [2]. These proteins can be further classified into random coils, molten, and pre-molten globules depending on the amount of transient secondary and tertiary structural elements and compaction [3]. Through their dynamic nature, IDPs can fulfill versatile roles in cellular processes [4,5]. They can serve as flexible linkers between domains or participate in molecular recognition [6]. By binding their specific partners, IDPs can undergo disorder-to-order transitions and acquire specific conformations. In addition, their structural states are influenced by factors such as pH, temperature, and post-translational modifications [7,8]. These transitions enable IDPs to modulate protein-protein interactions, enzymatic activity, and gene regulation [9]. Recently, IDPs have emerged to play important roles in driving or regulating the formation of membraneless organelles through liquid-liquid phase separation as well [10]. Altogether, IDPs are crucial for many signaling and regulatory processes in complex biological systems [11].

In the last two decades, a growing number of IDPs have been identified by various methods. One of the main sources of information about disordered proteins is the Protein Data Bank (PDB) [12]. Although the primary goal of PDB is to collect the structural coordinates of proteins, indirectly it can also indicate the presence of disorder as unresolved regions in structures determined by X-ray crystallography or cryo-electron microscopy or as high mobility segments by NMR. In many cases, IDP regions are defined based on sensitivity to proteases, increased molecular volumes, or a lack of secondary structure elements [13]. However, many of these techniques have some caveats. The complete characterization of IDPs requires a combination of different techniques centered around NMR measurements. The main resource for disordered proteins is the DisProt database [14]. Entries are collected from the literature by a community effort in order to annotate the specific regions and the applied methods. The DisProt database also gathers information about the corresponding function using specific ontology terms [14]. Currently, the database contains 2649 entries. Despite a steady increase, these cases represent only a tiny sample of disordered proteins encoded by the genomes of various organisms, especially in higher eukaryotes [15]. At this scale, computational prediction methods play a crucial role in the characterization of IDPs.

Until today, more than 100 different disordered prediction methods have been developed [16,17]. Ultimately, these all exploit the characteristic differences in the sequence features of ordered and disordered proteins. In general, disordered proteins are depleted in hydrophobic amino acids and enriched in polar residues [18]. Remarkably, a simple charge-hydropathy plot can separate structured proteins from intrinsically disordered proteins relatively well [3]. However, most practical applications demand a position-specific prediction of protein disorders. A wide range of principles have been applied for this task, including simple biophysical approaches as well as highly complex deep learning techniques that require millions of parameters [19]. One of the methods commonly employed by the scientific community is IUPred, which utilizes simple biophysical principles [20]. A more recent approach is based on the AlphaFold2 (AF2) method, which achieved breakthrough performance for structure prediction [21]. Although AlphaFold2 was not originally intended for identifying disordered regions, previous studies have demonstrated a strong correlation between its predicted local distance difference test (pLDDT) scores and disorder propensity [22,23]. In order to assess the state of the art in the field of disorder prediction, the CAID (Critical Assessment of Protein Intrinsic Disorder) challenge was established. The evaluations were based on a newly annotated subset of the DisProt database and showed that overall methods are sufficiently mature to be useful, but substantial room for improvement remains [16].

In this work, we conducted a comparative analysis of disorder prediction methods on the DisProt database. We focused on two approaches: IUPred and the pLDDT scores based on AF2 models. The two methods are based on completely different principles (biophysical vs. deep learning), and another crucial difference between them is that IUPred predictions are based on a single sequence while AF2 relies on multiple sequence alignments. However, in both cases, the training set contained only structured proteins, which enables us to test their performance on the complete DisProt protein dataset. In order to gain insights into the hidden biases and limitations of these methods, we test how well they agree and disagree in predicting experimentally characterized disordered regions and their subsets associated with diverse experimental methods and functional roles.

## 2. Materials and Methods

### 2.1. Disorder Dataset

The disordered dataset was obtained from the DisProt database (DisProt 2022/12) [14]. Disordered regions were defined based on the annotation of disordered structural states; additional terms were used for functional analysis. The disordered subset was filtered for redundancy using the CD-HIT tool [24] with identity set to 40% and word length to 2. The filtered database holds 4193 regions with disordered structural state annotations. These annotations correspond to 1922 proteins in 311 organisms and span 336,771 residues. However, as entries in DisProt may overlap, after merging each overlapping entry, this accounts for 1967 regions spanning 130,678 residues. 

### 2.2. Proteomes

We selected 55 model organisms for which precalculated AlphaFold2 predictions were available [21,25]. These included 16 bacteria, 31 eukaryotic, and 1 Archean proteome. In order to get a more complete coverage of Archean species, seven additional Archean species were added that were present in the QFO database (https://www.ebi.ac.uk/reference_proteomes/ accessed on 17 July 2023) (version 2023_03) [26], resulting in eight Archean organisms in total.

### 2.3. Prediction Methods

For the disorder prediction, we employed the most recent iteration of IUPred (v3) with default (medium smoothing) parameters [20]. We downloaded the pre-calculated AF2 models from the FTP server (https://ftp.ebi.ac.uk/pub/databases/alphafold accessed on 7 August 2023) (version 4) and utilized the pLDDT scores for disorder prediction. Secondary structure prediction was carried out using DSSP (version 4.0.4) [27] integrated into Biopython (v 1.81).

### 2.4. Evolutionary Conservation

To calculate the sequence conservation-based evolutionary classification of DisProt regions, multiple sequence alignments were downloaded from the HoTIDP database [28] using the rest API function (parameters: aln_method: mafftD, aln_type: region, dp_identity: 0.6, fullseq_identity: 0.6, regionseq_identity: 0.6, nd_full_cutoff: 0.6, nd_region_cutoff: 0.6). Only regions of mammalian organisms were included in the evolutionary classification. In total, 1296 region alignments were downloaded. The ortholog sequences were classified into three main evolutionary levels according to the UniProt taxonomic lineage: Vertebrata, Metazoa, and Unicellular. A region was considered conserved at a given evolutionary level if it was aligned with at least three orthologous sequences.

## 3. Results

### 3.1. Agreement and Disagreement between Disorder Predictions

In order to assess the level of agreement among different methods for predicting disorder in Intrinsically Disordered Proteins (IDPs) and Intrinsically Disordered Regions (IDRs), we conducted an analysis using the DisProt database and two widely used methods: IUPred and AlphaFold (AF2). We evaluated their performance on the non-redundant set of annotated disordered segments from the DisProt database, which contained 4193 regions in 1922 proteins spanning 336,771 residues. Here, we focused on non-overlapping, long regions that contained at least 30 residues. For the pLDDT scores, we used the 0.7 cutoff (residues below 0.7 are predicted to be disordered), consistent with prior measurements [23]. This way, pLDDT scores predicted 73% of residues as disordered. For IUPred, we set the cut-off value at 0.425 (residues above this cutoff are predicted to be disordered). At this cutoff value, IUPred predicted the same amount of residue as disordered.

**Figure 1:**
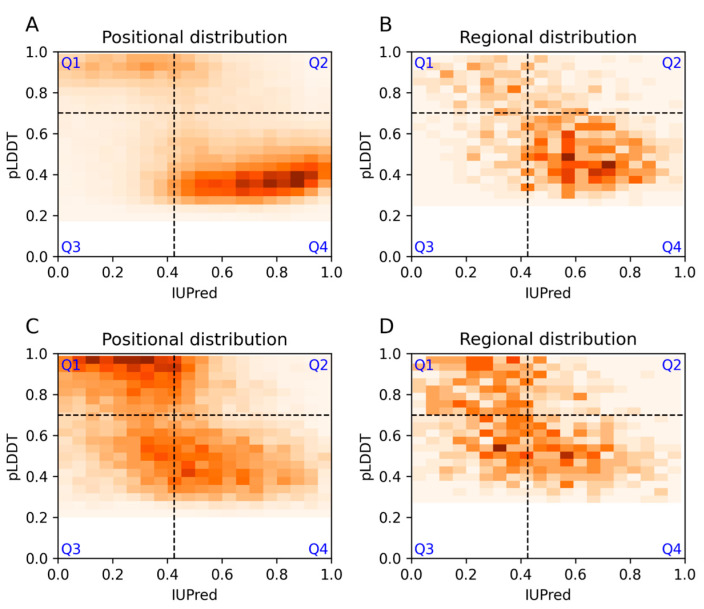
IUPred and AlphaFold2 pLDDT scores for each residue (**left**) and region (**right**) in DisProt with disordered structural state annotation are represented as a density heatmap. (**A**,**B**) Prediction scores for long disordered regions. (**C**,**D**) Scores for short disordered regions. Darker colors correspond to more densely populated areas.

For the purpose of a more detailed analysis, we introduced here a representation that plots the agreement and disagreement between the two methods and the DisProt annotations. Accordingly, we established four categories: Q1: both methods predict order; Q2: AF2 predicts order and IUPred predicts disorder; Q3: AF2 predicts disorder and IUPred predicts order; Q4: both methods predict disorder in agreement with the DisProt annotations and show the density heatmap of the scores within the different quadrants. Figure 1A shows the results for residues of long disordered regions, while a projected density distribution can be found in Supplementary Figure S2. The Q4 region covered 61% of the positions. Due to the selected cutoff values, Q2 and Q3 contained similar amounts, corresponding to 12% of the residues (Table 1). Notably, the Q1 category, when both methods predicted order, contained 15% of the cases.

We also considered the average predicted values for each extended, non-overlapping disordered region in DisProt instead of individual residues (Figure 1B). The observed trends remained similar at the level of regions, with some minor deviations. In this case, the Q4 region was slightly larger (64%), and the number of regions where AF2 alone disagreed with the disorder annotation was slightly lower (9% in Q2), as opposed to the 12% for IUPred detected in the Q3 quarter. These results indicate that in a significant portion of the cases (15%), both methods contradicted experiment annotations in DisProt at both the level of residues and regions (Table 1). 

We also generated a similar representation for short, disordered segments (less than 30 residues). The results show that this category was more challenging for both methods, but especially for IUPred (Figure 1C). While AF2 could predict 59% of residues correctly, IUPred recognized only 48% of disordered residues, and both methods agreed on only 35% of the annotated positions. However, in 28% of the cases, they both disagreed with DisProt annotations at the residue level, with largely similar values at the region level (Figure 1C,D). Interestingly, many of the residues within the short DisProt regions are predicted as ordered with high confidence. 

There are a handful of examples where a protein can have different conformational states induced by experimental conditions, including metamorphic and moonlight proteins. We analyzed a list of previously published proteins that exhibit this behavior and found that they tend to show more order in terms of pLDDR and IUPred score as well as overall compared to DisProt (Supplementary Figure S1) [29].

Overall, these results confirm earlier observations that there are characteristic differences between short and long disordered regions [30]. As long, disordered regions are more often associated with additional features, especially functional annotations, we focused on this subset in the rest of the manuscript. 

### 3.2. Ontology Terms

**Figure 2:**
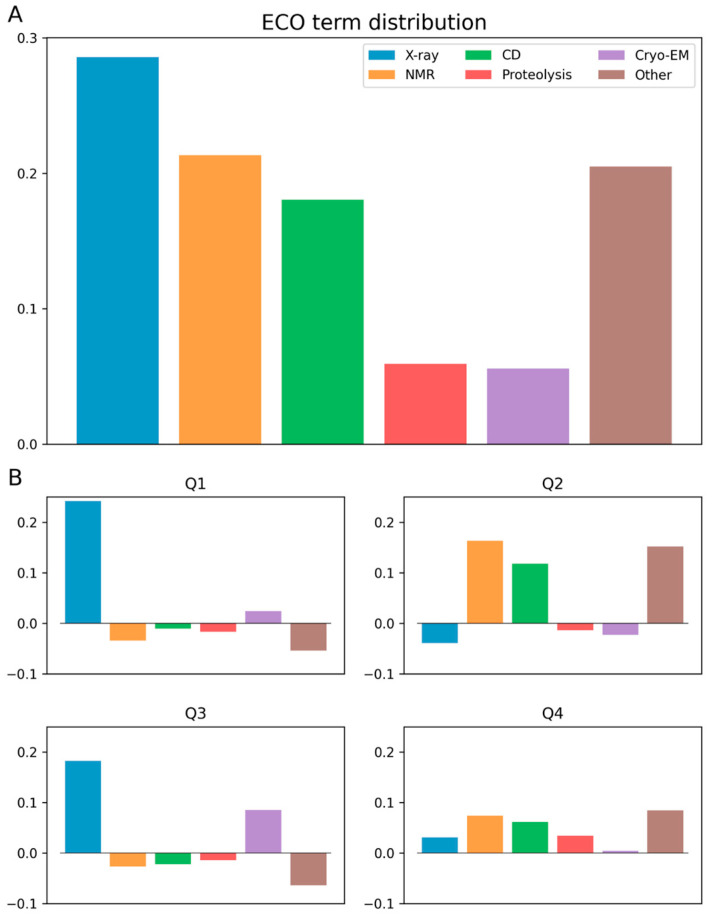
ECO IDs of experimental methods used in articles referenced by the DisProt annotation for each quartile. Terms with less than ten occurrences are omitted. (**A**) ECO term distribution for long DisProt regions. (**B**) ECO term distribution broken down into quadrants with respect to the distribution shown in Part A.

Next, we analyzed the type of experimental method that underlies the annotation of a given region based on the associated ECO (Evidence and Conclusion Ontology) terms [31]. Even for long disordered regions, the most common source of annotations is X-ray crystallography (ECO:0006220), in agreement with earlier observations that long disordered regions are now common in the PDB [32]. This method is followed by NMR spectroscopy (ECO:0006165), CD spectroscopy (ECO:0006204), cryo-EM (ECO:0006224), and proteolytic assays (ECO:0007691) (Figure 2A). Using the previously applied representation, we tested which methods were overrepresented and underrepresented in the different scenarios of agreement and disagreement between predictions and annotations. The results shown in Figure 2B indicate that in the Q1 quadrant, X-ray crystallography-based annotations were largely enriched. Based on the Q3 quadrant, disordered regions annotated with cryo-EM terms also seem to be more challenging for IUPred. In contrast, AF2 more often mispredicts regions annotated based on NMR, CD, or other techniques. 

**Figure 3:**
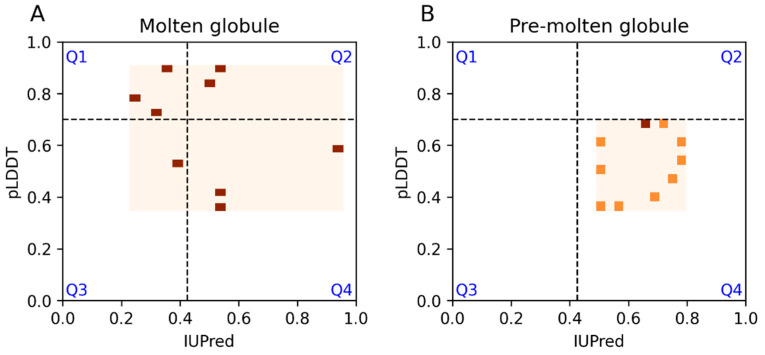
IUPred and AF2 pLDDT scores for DisProt regions with molten globule and pre-molten globule terms. Darker colors correspond to more densely populated areas.

DisProt has also introduced functional and structural ontology terms for intrinsically disordered regions known as IDPOs [14]. One of the main categories is the disordered structural state, which contains more specific child terms such as molten globule and pre-molten globule states. There are 9 and 11 regions associated with these terms in the filtered dataset, respectively. While all of the pre-molten globules occur in the Q4 quarter, nearly half of the annotated molten globules occur within the Q1 and Q2 regions (Figure 3A,B). This indicates that, to some extent, IUPred, but especially AF2, predicts a significant portion of these cases as ordered.

**Figure 4:**
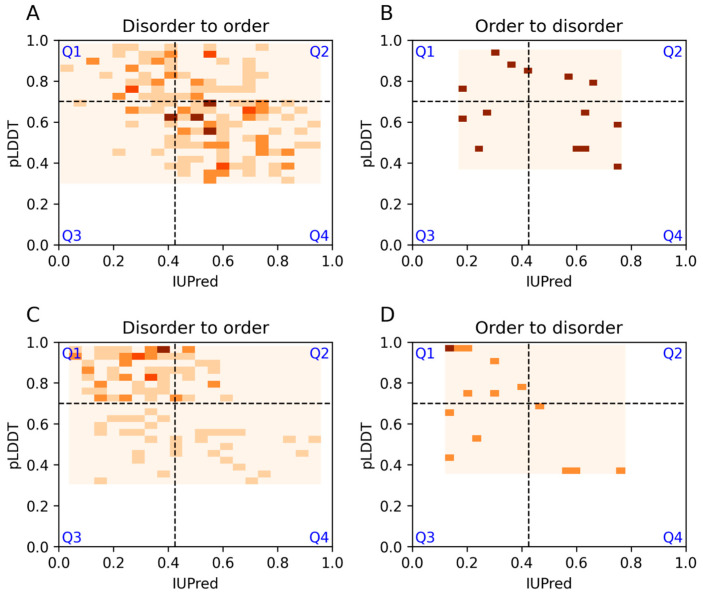
IUPred and AF2 pLDDT scores for DisProt regions with disorder-to-order and order-to-disorder structural transition IDPO terms. (**A**,**B**) Prediction scores for long disordered regions. (**C**,**D**) Scores for short disordered regions. Darker colors correspond to more densely populated areas.

Another major IDPO category is structural transition, in which the most common term is disorder-to-order transition. Additional child terms were not analyzed separately due to the limited number of examples. Predictions for regions with structural transitions generally agree well with each other. However, many of these examples are predicted as ordered by both methods (Figure 4A). AF2 predicted more additional orders compared to IUPred (Q2 vs. Q3). As many disordered regions with this annotation are short, we also looked at these examples separately. These shorter regions were mostly predicted as ordered by both methods (Figure 4C). Entries could also be annotated to undergo an order-to-disorder transition, although this term is used less frequently. Long disordered regions with this annotation do not show a preference for any of the quadrants, but shorter segments favor the Q1 region (Figure 4B,D). It is worth noting that annotations for structural transitions are likely to be incomplete, as around half of all DisProt regions contain corresponding PDB structures.

**Figure 5:**
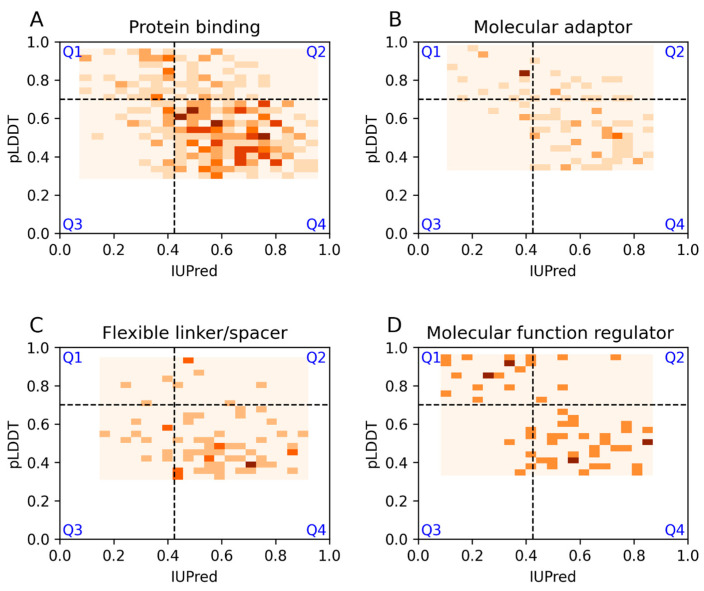
IUPred and AF2 pLDDT scores for long DisProt regions with the most frequently used ontology terms. (**A**–**D**) Prediction scores for Protein binding, Molecular adaptor, Flexible linkers/spacers, Molecular function regulator ontology terms respectively. Darker colors correspond to more densely populated areas.

The functional role of disordered regions is characterized by a combination of IDPO and GO terms [14]. Plots are shown here for the most frequently used ontology terms, corresponding to protein binding (GO:0005515), molecular adaptor activity (GO:0060090), flexible linker/spacer (IDPO:00502), and molecular function regulator (GO:0098772) (Figure 5). The profile for the protein binding category is similar to the one corresponding to disorder-to-order transition, as expected, with the majority of cases occupying the Q4 area. Examples of molecular adaptor function mostly occur within the Q4 and Q1 regions, with very few cases within the Q3 quadrant. Complementing categories related to molecular recognition, the flexible linker/spacer category is part of the entropic chain function. For this type of functional term, most examples are located within the Q4 quadrant, and there are very few examples within Q1 regions. The slightly increased number of examples within Q3 compared to Q2 indicates that linkers in general are better recognized by AF2. In contrast, AF2 and IUPred largely agree for cases with molecular function regulator activity, but many of these examples are predicted as ordered by both methods.

### 3.3. Secondary Structure Preferences

**Figure 6:**
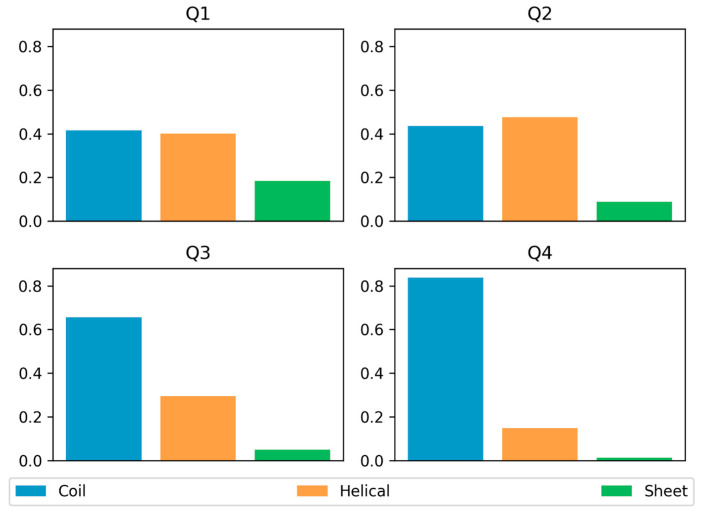
Secondary structure distribution of proteins in DisProt with disordered structural state predicted by AlphaFold for each quartile.

We also examined secondary structure elements in the AlphaFold-predicted structures of proteins in DisProt using DSSP [27] (Figure 6). In general, only a small or no amount of regular secondary structure elements are expected within disordered regions. In agreement, the fourth quadrant showed only limited tendencies towards α-helical and β-sheet structures. The Q3 region showed a slightly higher amount of predicted regular secondary structure elements, but coil structures still dominated with over 60 percent. AF2 predictions with high pLDDT scores were correlated with an increased amount of regular secondary structure elements, especially α-helices. This was true not only for Q1 but also for the Q2 quadrant, in which α-helices were the most commonly predicted structural elements. This indicates a strong bias for AlphaFold2 to predict α-helical elements within disordered regions. Another key observation is that AF2 predicts significantly lower amounts of β-sheets for regions with low pLDDT scores, while α-helices are more prevalent in regions with high confidence.

### 3.4. Sequence Conservation

**Figure 7:**
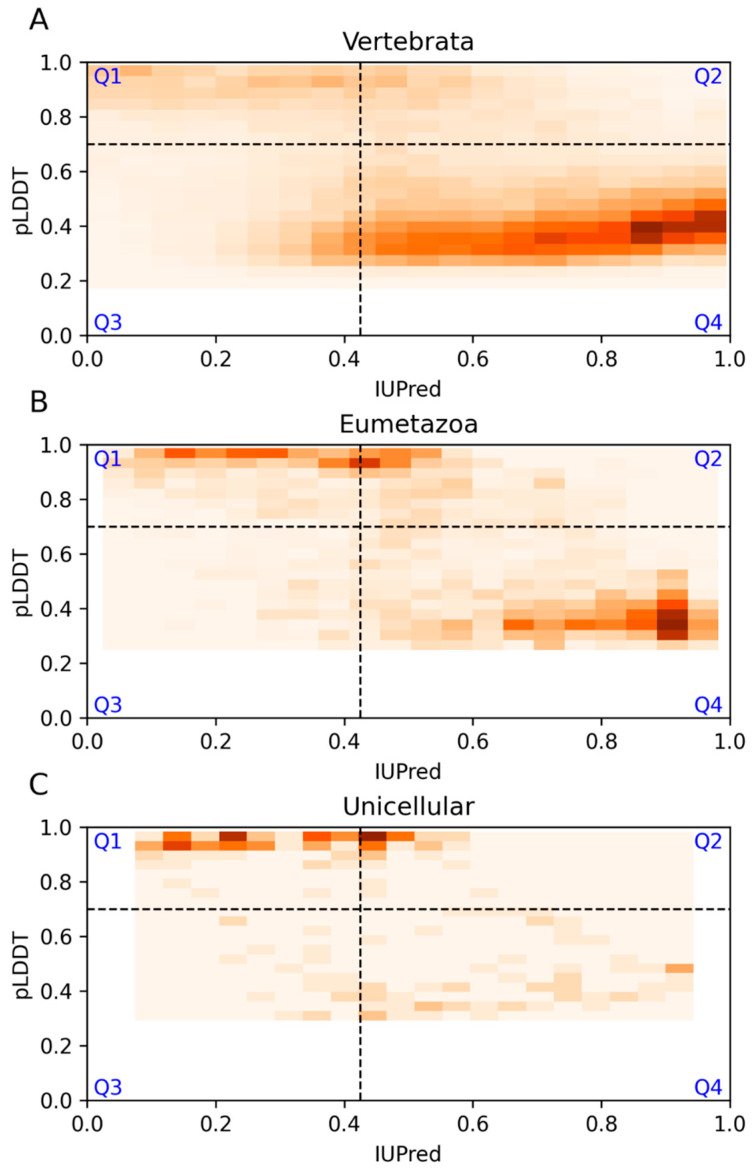
IUPred and AF2 pLDDT scores for long DisProt regions classified by evolutionary sequence conservation. (**A**–**C**) sections represent Vertebrata, Eumetazoa and Unicellular organisms respectively. Darker colors correspond to more densely populated areas.

We also assessed the level of agreement between AF2 and IUPred based on different levels of evolutionary sequence conservation calculated from multiple sequence alignments (MSAs) of orthologs. For this, we focused on sequences from mammalian species containing long, disordered regions. The MSAs were collected from the HoTIDP database, which stores precalculated reference alignments of DisProt entries [28]. Based on the MSAs, the level of conservation was classified as Vertebrata, Metazoa, or Unicellular (methods) for each DisProt region. 1225 examples could be traced back to the Vertebrata level only, while 51 examples showed a more ancient evolutionary origin at the Eumetazoa level, and 20 examples could be traced back to the level of unicellular organisms. Most of the cases of Vertebrate origin were predicted to be disordered by both methods (Figure 7A). Examples that could be traced back to Eumetazoa clustered into two regions, one with a high IUPred/low pLDDT score and one with a high pLDDT score, some of which had high IUPred scores (Figure 7B). Interestingly, our results showed that in the case of the Unicellular subset, 60% of the positions annotated as disordered in DisProt were predicted as ordered by AF2 (Unicellular Q1 plus Q2) (Figure 7C) (Table 2). This result showed that cases with more conserved orthologs tend to be predicted as ordered regions by AF2. This tendency can be explained by the fact that the key input to AF2 is multiple sequence alignment, and the larger number of evolutionarily related sequences promotes the prediction of a structured state. On the other hand, predictions based on IUPred are expected to be less affected by this conservation-based bias. Accordingly, the percentage of residues in the Q3 region slightly decreases for proteins for larger evolutionary conservation, with only 9% of residues classified as disordered by IUPred alone (Unicellular Q3) (Table 2).

### 3.5. Model Proteomes

**Figure 8:**
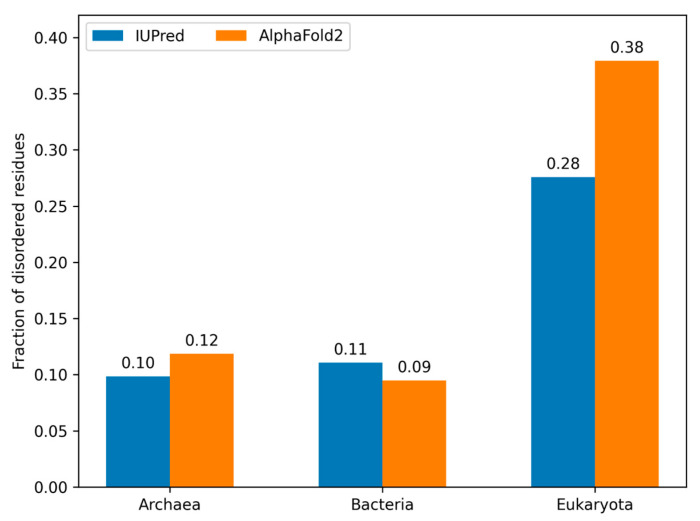
Distribution of positions predicted to be disordered by AF2 and IUPred at the eukaryote, bacteria, and Archaea evolutionary levels. The proteomes of the three main evolutionary levels are based on a total of 55 model organisms.

While the DisProt database serves as a great starting point, the number of known examples is still limited. In order to gain a better understanding of the discrepancies in disorder prediction methods, we analyzed the publicly available reference proteomes in the AlphaFold database, completed with Archean organisms from the QFO database (see Section 2). We collected IUPred predictions and the pLDDT score and calculated the percentage of disordered residues for bacteria, Archaea, and eukaryotic species using the same cutoff values as previously. There was no significant difference observed for bacteria and Archaea, with both methods predicting around 10% of residues as disordered. However, we observed a more significant difference in the case of eukaryotic proteomes. While AF2 predicted 38% of the residues as disordered in this set, IUPred only predicted 28% (Figure 8). 

### 3.6. Examples

One of the unexpected outcomes of our analyses is the relatively high proportion of residues when the IUPred and AF2 methods both predicted order for an annotated disordered region. In order to gain further insights, we took a closer look at some examples. The observed discrepancy can highlight cases with weak or erroneous experimental support. One such potential case is the DisProt entry DP02515 for the human CD166 antigen (Q13740), with region 246–583 annotated as disordered based on the PDB structure 5A2F. The authors stated that the regions “had been proteolytically cleaved prior to crystal growth”, therefore this region should not have been indicated as having missing residue coordinates from the PDB structure. The longest ambiguous case corresponds to DP02513 (Q05022) annotated according to the cryo-EM-based structural model with the PDB code 5WYJ [33]. Over 1400 residues are annotated as disordered at the N-terminal that are missing from multiple structures. However, this region contains repeating units of S1 domains, which are common in RNA-binding proteins and are assumed to fold into five-stranded antiparallel β barrel structures [34]. Therefore, it needs further experimental confirmation whether this region is indeed disordered, or if only its relative orientation varies but disorder is limited to a much shorter linker region. 

Another common scenario is when both AF2 and IUPred predict order for disordered regions, which corresponds to disordered-to-order transitions. One example of this is the peptide fragment from 248 to 286 of prostatic acid phosphatase (PAP) (DP00628). This peptide is a naturally occurring 39 amino acid fragment from PAP that significantly enhances the rate of HIV infection [35]. The monomeric peptide PAP248-286 was shown to be predominantly disordered in solution. However, NMR spectroscopy in SDS micelles, which serve as a model membrane system, has shown that the core of the peptide (262–270) adopts a short helical region during a disordered-to-ordered transition when bound to the surface of the micelle, while the N- and C-termini remain highly flexible [35]. Both AF2 and IUPred predict PAP248-286 as an ordered region. This is in agreement with the behavior of the peptide in the context of the complete protein, which adopts a well-defined structure [36].

**Figure 9:**
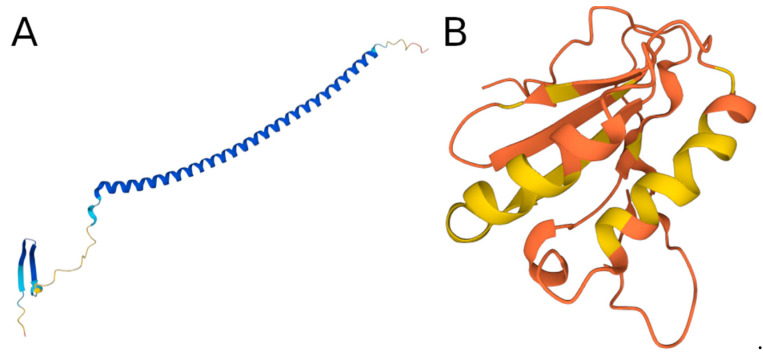
(**A**) AlphaFold2 structure of the human Stathmin protein (P16949). (**B**) AlphaFold2 structure of the human putative uncharacterized protein UNQ6493/PRO21345 (Q6UXR8). Both structures are colored according to pLDDT scores (red corresponds to lower confidence, while blue indicates higher confidence).

One notable feature of AF models for disordered regions is that they predict α-helical regions with high pLLDT scores even within experimentally verified disordered segments. One example of this is the human Stathmin protein, which is involved in the regulation of the microtubule and has been shown to be disordered between residues 116 and 168 [37]. However, the AlphaFold2 structure of the protein shows strong helical preference with high confidence in this region (Figure 9A).

Our results also showed that AF2 tends to predict intrinsically disordered regions as ordered in the case of strong evolutionary sequence conservation. However, IUPred is able to predict these cases as disordered. An example is the region (DP00393) from nuclear cap-binding protein subunit 2 (CBP20) of the Cap Binding Complex (CBC), which is extremely conserved not only in vertebrates and eumetazoans but also in unicellular organisms. This results in a large number of homologous sequences in the AF2 MSA input, which can promote order prediction. In contrast, IUPred predicts the region as disordered, which is consistent with PDB structures where the region has missing residue coordinates in an unbound state (1H2V, 1N54). There are additional PDB structures where CBP20 is in complex with a ligand or a partner (1H2T, 1H2U, 5OO6) and the region adopts a structure very similar to the AF2 model, suggesting that AF2 prediction is influenced by these structures. This suggests that not only the disordered region itself but also its transition to an ordered state is evolutionarily conserved. However, AF2 is biased towards recognizing the ordered state, while IUPred predicts this region as disordered.

One of the surprising results of our analysis was that although IUPred and AF2 predict the same amount of disorder for DisProt and for Archeal and bacterial species, there are significantly more residues disordered in eukaryotic proteomes according to AF2 (Figure 8). An interesting case when AF2 predicts disorder but IUPred predicts order is the case of the putative uncharacterized protein UNQ6493/PRO21345 (Q6UXR8) (Figure 9B). The predicted structure of AlphaFold2 shows a generally well-structured protein, which is in agreement with the IUPred prediction of order; however, the pLDDT score is very weak for all residues, which would indicate the presence of disorder. There are further studies needed to understand the origin of this proteome-level bias.

## 4. Discussion

The DisProt database is the largest collection of experimentally verified disordered protein regions [14]. Entries are supported by experimental evidence and, if available, complemented with specific annotation terms related to structural states, transitions, and functional aspects. As a result, the DisProt database provides a comprehensive representation of various IDP subclasses—or flavors—of disorder [38]. These flavors can depend on the length of the disordered region or the experimental method used for their identification [1,30]. A subset of IDRs were suggested to exist in a molten globule or premolten globule structural state or undergo a disorder-to-order or order-to-disorder transition depending on environmental conditions or binding to specific partners [2]. The detailed structural properties of IDPs have been associated with various functions and their regulation. Given the heterogeneous nature of protein disorder, one central question is how disorder prediction methods are able or limited in their ability to capture the different flavors of disorder. Recent large-scale assessments of disorder prediction methods indicated that there is still a need for further improvements [16,39]. However, these evaluations give little clue about the main biases and limitations of individual methods. In order to shed light on this, we utilized the DisProt database and compared two disorder prediction methods that were trained on different principles, IUPred and AF2, and explored the agreements and disagreements in their prediction results.

Our results revealed that, considering residues located within long regions from the DisProt database, IUPred and AF2 provided consistent predictions in 79% of the cases. However, for 15% of these cases, both methods predicted order. Various factors could contribute to the disagreement between disorder annotations and computational predictions. One source could be weak experimental support, especially based on methods that do not have a residue-level resolution, such as proteolytic assays or CD spectroscopy. Disordered regions defined by these methods could contain ordered domains in addition to disordered regions. In addition, the class of molten globules, which can be viewed as an intermediate state between full order and disorder, is also mostly predicted as ordered. A large group of additional cases that are consistently predicted as ordered, especially in the case of shorter segments, correspond to regions undergoing disorder-to-order transitions. Many of these examples can exist in both an ordered and a disordered state, and they can often be found by contrasting regions with and without known coordinates from PDB structures. This is supported by the fact that for this category, X-ray crystallography techniques and, to a lesser extent, cryo-EM structures are overrepresented. From a functional point of view, molecular function regulators were strongly associated with disordered regions predicted as ordered by both methods.

Our analysis also indicates that there are major differences in how the two methods capture the flavors of disorder represented in DisProt. For example, IUPred can recognize short, disordered regions much less effectively compared to AF2; however, these differences largely diminish for longer disordered regions. While IUPred encounters more difficulties with disordered regions defined based on X-ray crystallography, the challenging cases for AF2 are more likely to come from experimental evidence based on NMR or CD. For regions annotated as linkers in the DisProt database, IUPred predicts more order. In contrast, AF2 predicts order for a larger portion of regions involved in protein binding and for molecular adaptors. These results demonstrate that discrepancies between the two predictions can be linked to functional information, which could be exploited for the development of specialized approaches for the recognition of functional regions, for example, binding regions or linker segments [40,41,42]. One of the observed features of AF2 is that α-helical segments are significantly overrepresented in the predicted structures for the disordered regions. In general, regions that show stronger evolutionary conservation tend to be predicted as more ordered, especially by AF2. We also showed that these biases manifest strongly at the level of proteomes, with AF2 predicting a larger proportion of eukaryotic genomes to encode disordered proteins.

In conclusion, in this work, we compared two disorder prediction methods, IUPred and AF2, in order to shed light on their ability to capture the diversity of disorders using the DisProt annotations. Our analysis revealed major differences between IUPred and AF2 in capturing the various flavors of disorder, with each method exhibiting unique strengths and limitations based on region length, experimental evidence, and functional annotations. We also show that these biases can also be linked to evolutionary conservation, taxonomical origin, and structural preferences. Overall, these types of analyses can be extended to other methods and might provide valuable insights into the strengths and limitations of disorder prediction methods, paving the way for approaches with improved performance.

## Figures and Tables

**Table 1:** Distribution of long and short region positions by prediction scores across the four quadrants.

	Long		Short	
	Residues	Regions	Residues	Regions
Q1	17,444 (15%)	139 (15%)	5240 (28%)	290 (29%)
Q2	13,290 (12%)	85 (9%)	2259 (13%)	106 (10%)
Q3	13,043 (12%)	119 (12%)	4444 (24%)	264 (26%)
Q4	68,401 (61%)	603 (64%)	6557 (35%)	361 (35%)
Sum	112,178	946	18,500	1021

**Table 2:** Distribution of DisProt region positions in the four quadrants at the three evolutionary levels.

	Vertebrata	Eumetazoa	Unicellular
Q1	1150 (13%)	578 (22%)	83 (40%)
Q2	9925 (12%)	480 (19%)	41 (20%)
Q3	10,705 (13%)	144 (6%)	18 (9%)
Q4	52,222 (62%)	1378 (53%)	63 (31%)
Sum	74,002	2580	205

## Data Availability

The data presented in this study are available in this article.

## Supplementary Materials

The following supporting information can be downloaded at: https://www.mdpi.com/article/10.3390/biom13101442/s1, Figure S1: IUPred and AlphaFold2 pLDDT scores for each residue (left) and region (right) of metamorphic and moonlight proteins represented as a density heatmap; Figure S2: Projected density distribution or IUPred and AlphaFold2 pLDDT scores for each residue (left) and region (right) in DisProt with disordered structural state annotation. Table S1: IDPO and GO Functional annotations in the DisProt database.
