# Supplementary material for: The Origin of Discrepancies between Predictions and Annotations in Intrinsically Disordered Proteins
Source: Biomolecules. 2023 Sep 25;13(10):1442. doi: 10.3390/biom13101442 (PMC10604070; doi:10.3390/biom13101442)
Supplement: Supplementary file 1 [file biomolecules-13-01442-s001.zip › biomolecules-2575230-supplementary.pdf]

Figure S1: IUPred and AlphaFold2 pLDDT scores for each residue (left) and region (right) of metamorphic and moonlight proteins represented as a density heatmap

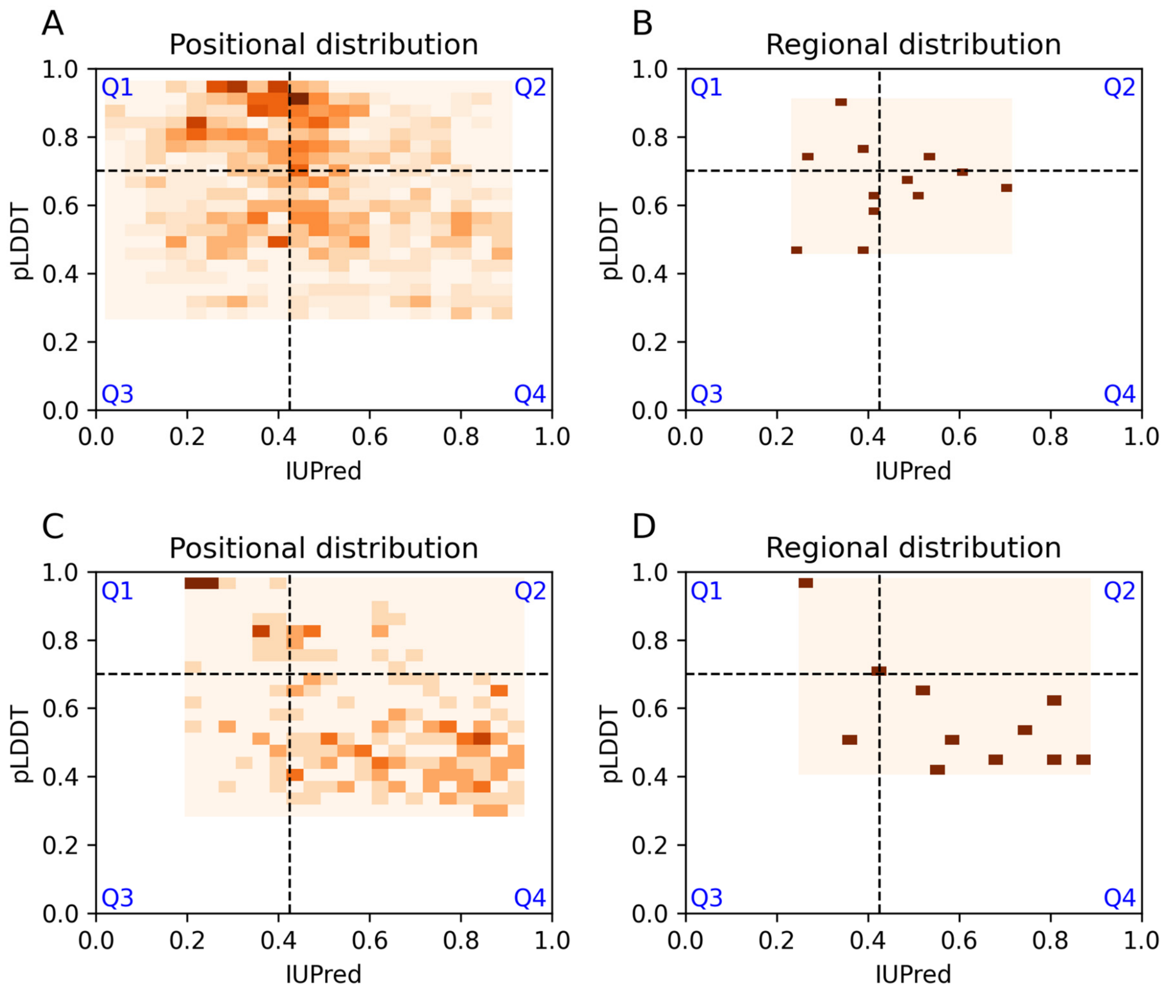

Figure S2: Projected density distribution or IUPred and AlphaFold2 pLDDT scores for each residue (left) and region (right) in DisProt with disordered structural state annotation.

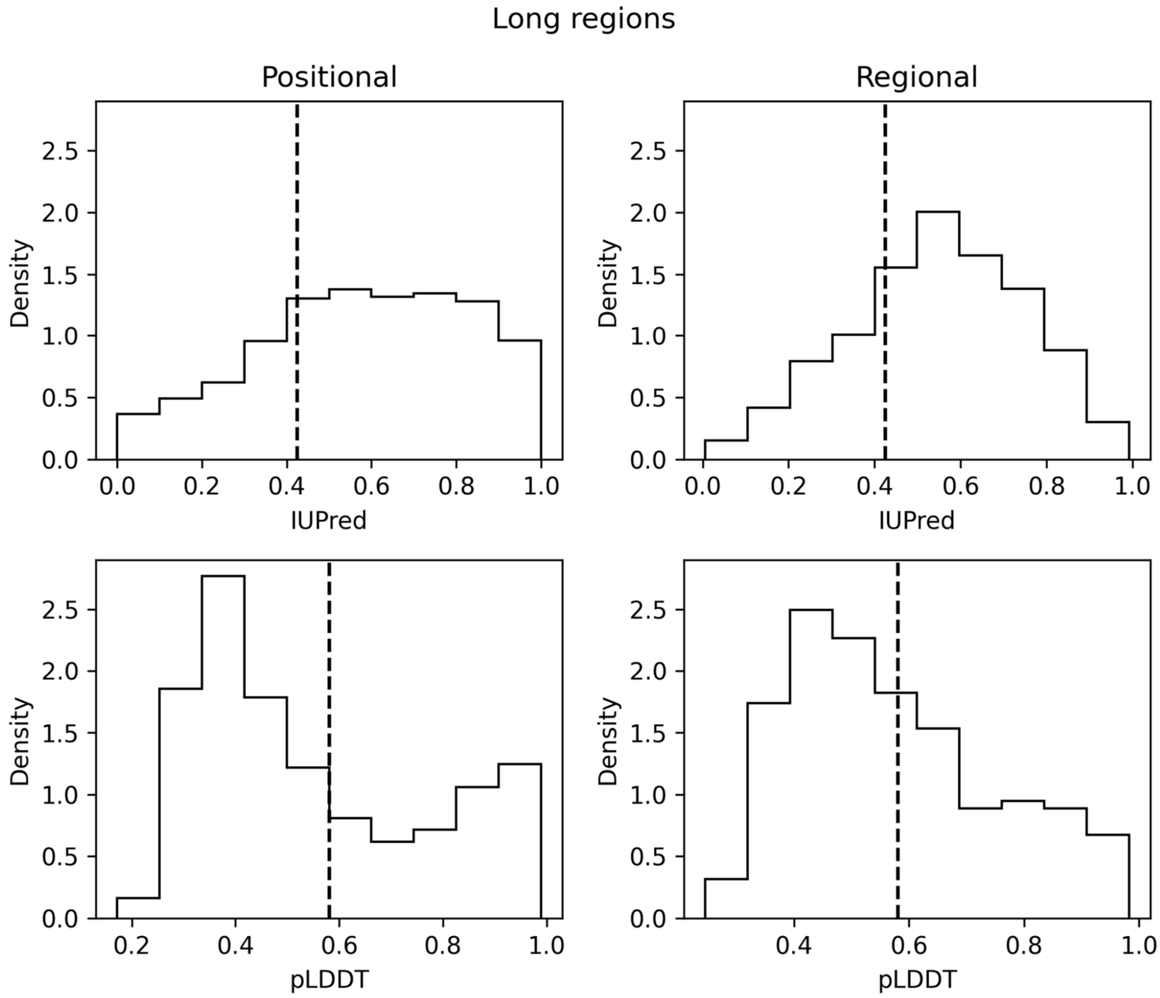

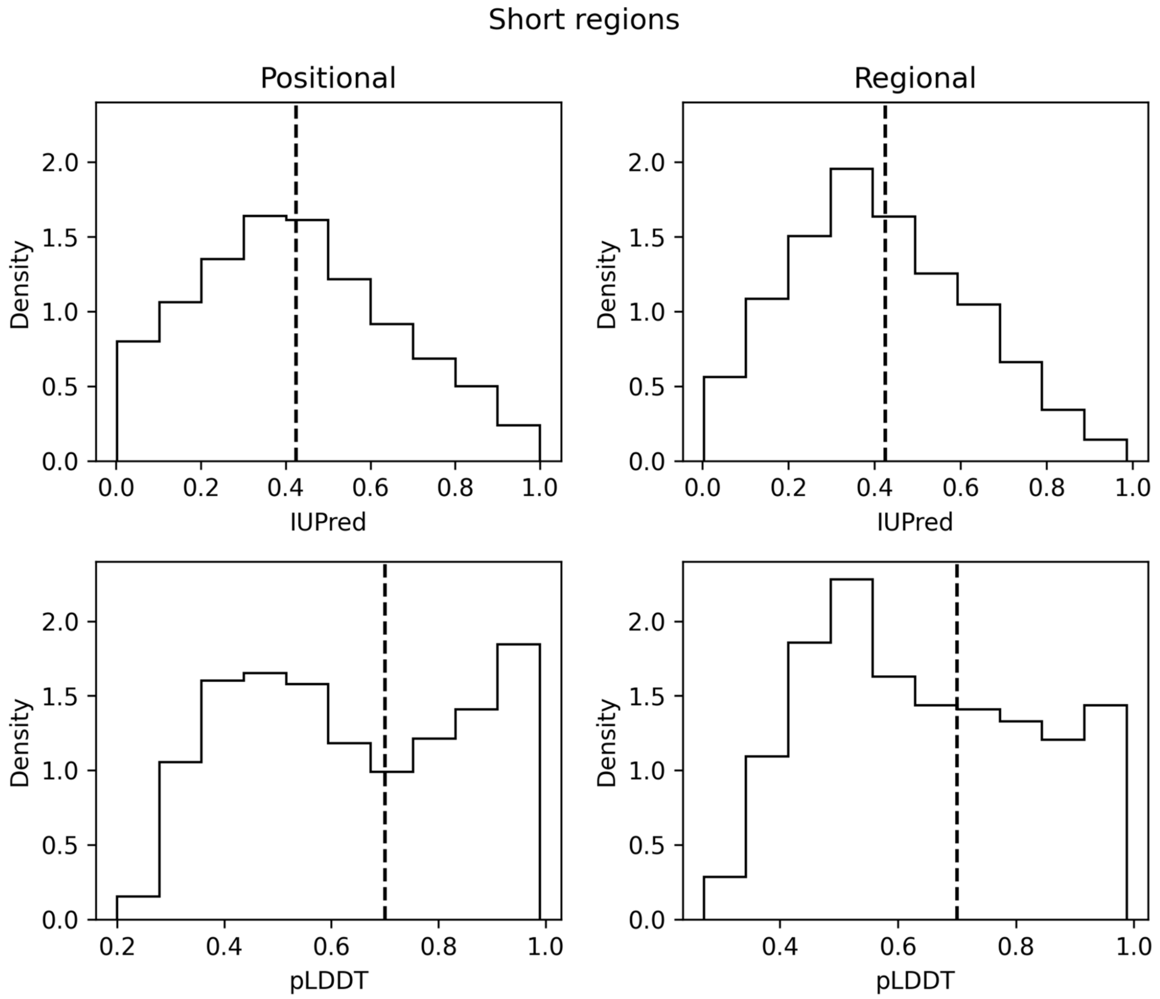

Table S1: IDPO and GO Functional annotations in the DisProt database

Q1	IDPO:00076	disorder	474
Q1	GO:0005515	protein binding	108
Q1	IDPO:00050	disorder to order	107
Q1	GO:0098772	molecular function regulator	39
Q1	IDPO:00502	flexible linker/spacer	24
Q1	GO:0060090	molecular adaptor activity	23
Q1	IDPO:00056	order to disorder	14
Q1	IDPO:00079	order	12
Q1	GO:0036094	small molecule binding	11
Q1	GO:0008289	lipid binding	10
Q1	GO:0003676	nucleic acid binding	10
Q1	IDPO:00025	phosphorylation display site	8

Q1	IDPO:00501	entropic chain	7
Q1	GO:0003677	DNA binding	7
Q1	GO:1990000	amyloid fibril formation	5
Q1	GO:0003723	RNA binding	5
Q1	GO:0140678	molecular function inhibitor activity	4
Q1	IDPO:00077	molten globule	4
Q1	GO:0140677	molecular function activator activity	4
Q1	GO:0140313	molecular sequestering activity	4
Q1	GO:0051179	localization	4
Q1	IDPO:00033	limited proteolysis display site	3
Q1	GO:0061912	selective autophagy	3
Q1	GO:0097351	toxin sequestering activity	3
Q1	GO:0043232	intracellular non-membrane-bounded organelle	2
Q1	GO:0001849	complement component C1q complex binding	2
Q1	IDPO:00053	molten globule to order	2
Q1	IDPO:00055	pre-molten globule to order	2
Q1	GO:0043167	ion binding	2
Q1	GO:0051238	sequestering of metal ion	2
Q1	GO:1900407	regulation of cellular response to oxidative stress	2
Q1	IDPO:00504	flexible C-terminal tail	2
Q1	GO:0044183	protein folding chaperone	2
Q1	GO:0046872	metal ion binding	2
Q1	GO:0003729	mRNA binding	2
Q1	GO:1905761	SCF ubiquitin ligase complex binding	1
Q1	GO:1900037	regulation of cellular response to hypoxia	1
Q1	IDPO:00508	self-assembly	1
Q1	IDPO:00032	palmitoylation display site	1
Q1	IDPO:00024	molecular recognition display site	1
Q1	GO:0005161	platelet-derived growth factor receptor binding	1
Q1	GO:0070734	histone H3-K27 methylation	1
Q1	GO:0005506	iron ion binding	1
Q1	GO:0071569	protein ufmylation	1
Q1	GO:1905906	regulation of amyloid fibril formation	1

Q2	IDPO:00076	disorder	228
Q2	GO:0005515	protein binding	76
Q2	IDPO:00050	disorder to order	59
Q2	GO:0060090	molecular adaptor activity	21
Q2	IDPO:00502	flexible linker/spacer	15
Q2	GO:0098772	molecular function regulator	12
Q2	GO:0140678	molecular function inhibitor activity	10
Q2	GO:0003677	DNA binding	8
Q2	GO:0003676	nucleic acid binding	7
Q2	IDPO:00025	phosphorylation display site	6
Q2	IDPO:00079	order	6
Q2	GO:1990000	amyloid fibril formation	6
Q2	GO:0051179	localization	5
Q2	GO:0008289	lipid binding	5
Q2	GO:0036094	small molecule binding	3
Q2	IDPO:00056	order to disorder	3
Q2	GO:0061912	selective autophagy	2
Q2	IDPO:00077	molten globule	2
Q2	GO:0001849	complement component C1q complex binding	2
Q2	GO:0031625	ubiquitin protein ligase binding	2
Q2	IDPO:00026	acetylation display site	2
Q2	IDPO:00027	methylation display site	2
Q2	GO:0003723	RNA binding	2
Q2	GO:0046872	metal ion binding	2
Q2	IDPO:00052	disorder to pre-molten globule	1
Q2	GO:1904030	negative regulation of cyclin-dependent protein kinase activity	1
Q2	GO:0045893	positive regulation of transcription, DNA-templated	1
Q2	GO:0008285	negative regulation of cell population proliferation	1
Q2	GO:0009968	negative regulation of signal transduction	1
Q2	GO:0010468	regulation of gene expression	1
Q2	GO:0003714	transcription corepressor activity	1
Q2	GO:0010507	negative regulation of autophagy	1
Q2	IDPO:00029	ubiquitination display site	1

Q2	GO:0005049	nuclear export signal receptor activity	1
Q2	IDPO:00504	flexible C-terminal tail	1
Q2	IDPO:00506	self-inhibition	1
Q2	GO:0097351	toxin sequestering activity	1
Q2	IDPO:00078	pre-molten globule	1
Q2	GO:0140693	molecular condensate scaffold activity	1
Q2	GO:0019843	rRNA binding	1
Q2	GO:0044183	protein folding chaperone	1
Q2	IDPO:00051	disorder to molten globule	1
Q2	IDPO:00058	order to pre-molten globule	1
Q2	GO:0140677	molecular function activator activity	1
Q2	GO:0140691	RNA folding chaperone	1
Q2	IDPO:00028	glycosylation display site	1
Q2	IDPO:00501	entropic chain	1
Q2	GO:0005506	iron ion binding	1
Q2	IDPO:00033	limited proteolysis display site	1
Q2	GO:1905906	regulation of amyloid fibril formation	1
Q3	IDPO:00076	disorder	454
Q3	GO:0005515	protein binding	73
Q3	IDPO:00050	disorder to order	38
Q3	IDPO:00502	flexible linker/spacer	37
Q3	IDPO:00025	phosphorylation display site	17
Q3	GO:0098772	molecular function regulator	17
Q3	GO:0060090	molecular adaptor activity	13
Q3	GO:0140678	molecular function inhibitor activity	10
Q3	GO:0140677	molecular function activator activity	7
Q3	IDPO:00056	order to disorder	6
Q3	GO:0008289	lipid binding	6
Q3	IDPO:00024	molecular recognition display site	5
Q3	GO:0003676	nucleic acid binding	5
Q3	GO:0051179	localization	5
Q3	IDPO:00506	self-inhibition	4
Q3	GO:0030246	carbohydrate binding	4

Q3	GO:0061912	selective autophagy	4
Q3	GO:1905761	SCF ubiquitin ligase complex binding	3
Q3	IDPO:00503	flexible N-terminal tail	2
Q3	GO:0044183	protein folding chaperone	2
Q3	IDPO:00027	methylation display site	2
Q3	IDPO:00026	acetylation display site	2
Q3	IDPO:00079	order	2
Q3	IDPO:00504	flexible C-terminal tail	2
Q3	GO:0140537	transcription regulator activator activity	2
Q3	GO:0036094	small molecule binding	2
Q3	GO:0003723	RNA binding	2
Q3	GO:0019835	cytolysis	1
Q3	GO:0005516	calmodulin binding	1
Q3	IDPO:00077	molten globule	1
Q3	GO:0001094	TFIID-class transcription factor complex binding	1
Q3	IDPO:00055	pre-molten globule to order	1
Q3	IDPO:00501	entropic chain	1
Q3	GO:0043232	intracellular non-membrane-bounded organelle	1
Q3	GO:0140693	molecular condensate scaffold activity	1
Q3	GO:1990000	amyloid fibril formation	1
Q3	IDPO:00032	palmitoylation display site	1
Q3	GO:0003677	DNA binding	1
Q3	IDPO:00028	glycosylation display site	1
Q3	GO:0000049	tRNA binding	1
Q3	GO:0006111	regulation of gluconeogenesis	1
Q3	GO:0045722	positive regulation of gluconeogenesis	1
Q3	GO:0140313	molecular sequestering activity	1
Q3	IDPO:00505	self-regulatory activity	1
Q3	GO:0098792	xenophagy	1
Q4	IDPO:00076	disorder	1194
Q4	GO:0005515	protein binding	304
Q4	IDPO:00050	disorder to order	122
Q4	IDPO:00502	flexible linker/spacer	111

Q4	GO:0060090	molecular adaptor activity	81
Q4	IDPO:00025	phosphorylation display site	59
Q4	GO:0098772	molecular function regulator	40
Q4	GO:0140678	molecular function inhibitor activity	24
Q4	GO:0003676	nucleic acid binding	23
Q4	GO:0140677	molecular function activator activity	21
Q4	GO:0003677	DNA binding	21
Q4	GO:0051179	localization	19
Q4	GO:0140693	molecular condensate scaffold activity	19
Q4	GO:0043167	ion binding	12
Q4	IDPO:00501	entropic chain	12
Q4	IDPO:00078	pre-molten globule	12
Q4	GO:0044183	protein folding chaperone	12
Q4	GO:0008289	lipid binding	10
Q4	GO:0003723	RNA binding	9
Q4	GO:0036094	small molecule binding	9
Q4	IDPO:00506	self-inhibition	9
Q4	GO:0031625	ubiquitin protein ligase binding	7
Q4	IDPO:00056	order to disorder	7
Q4	IDPO:00079	order	6
Q4	GO:0043232	intracellular non-membrane-bounded organelle	6
Q4	IDPO:00033	limited proteolysis display site	6
Q4	IDPO:00503	flexible N-terminal tail	5
Q4	IDPO:00504	flexible C-terminal tail	5
Q4	IDPO:00508	self-assembly	5
Q4	IDPO:00024	molecular recognition display site	5
Q4	IDPO:00028	glycosylation display site	4
Q4	IDPO:00029	ubiquitination display site	4
Q4	IDPO:00026	acetylation display site	4
Q4	IDPO:00052	disorder to pre-molten globule	4
Q4	GO:0061912	selective autophagy	4
Q4	GO:0002039	p53 binding	3
Q4	GO:1990000	amyloid fibril formation	3

Q4	IDPO:00077	molten globule	3
Q4	GO:0030246	carbohydrate binding	3
Q4	GO:0010508	positive regulation of autophagy	3
Q4	GO:0002151	G-quadruplex RNA binding	2
Q4	IDPO:00027	methylation display site	2
Q4	IDPO:00051	disorder to molten globule	2
Q4	IDPO:00060	pre-molten globule to molten globule	2
Q4	GO:0140691	RNA folding chaperone	2
Q4	GO:1990316	Atg1/ULK1 kinase complex	2
Q4	GO:0001223	transcription coactivator binding	2
Q4	GO:0140313	molecular sequestering activity	2
Q4	GO:0140486	zinc ion sequestering activity	2
Q4	GO:0046872	metal ion binding	2
Q4	GO:0005516	calmodulin binding	2
Q4	GO:0008285	negative regulation of cell population proliferation	1
Q4	GO:0001933	negative regulation of protein phosphorylation	1
Q4	GO:0001934	positive regulation of protein phosphorylation	1
Q4	GO:0140311	protein sequestering activity	1
Q4	GO:1990757	ubiquitin ligase activator activity	1
Q4	GO:0051168	nuclear export	1
Q4	GO:0140487	metal ion sequestering activity	1
Q4	GO:0003713	transcription coactivator activity	1
Q4	GO:0017025	TBP-class protein binding	1
Q4	GO:0071889	14-3-3 protein binding	1
Q4	GO:0008301	DNA binding, bending	1
Q4	GO:0043621	protein self-association	1
Q4	GO:0030308	negative regulation of cell growth	1
Q4	IDPO:00030	fatty acylation display site	1
Q4	GO:0051238	sequestering of metal ion	1
Q4	IDPO:00055	pre-molten globule to order	1
Q4	IDPO:00034	ADP-ribosylation display site	1
Q4	GO:0031396	regulation of protein ubiquitination	1
Q4	GO:0005506	iron ion binding	1

Q4	GO:0010506	regulation of autophagy	1
Q4	GO:0039521	suppression by virus of host autophagy	1
Q4	GO:0051647	nucleus localization	1
Q4	GO:0016301	kinase activity	1
Q4	GO:0042393	histone binding	1
Q4	GO:0070772	PAS complex	1
Q4	GO:0060341	regulation of cellular localization	1
Q4	GO:1905463	negative regulation of DNA duplex unwinding	1
Q4	GO:0051101	regulation of DNA binding	1
Q4	GO:0032092	positive regulation of protein binding	1
Q4	GO:0000049	tRNA binding	1
Q4	GO:0032508	DNA duplex unwinding	1
Q4	GO:0008270	zinc ion binding	1
Q4	GO:0140314	calcium ion sequestering activity	1
Q4	GO:0033596	TSC1-TSC2 complex	1
Q4	GO:0070585	protein localization to mitochondrion	1
Q4	GO:1903146	regulation of autophagy of mitochondrion	1
